# Robotic Surgery for Head and Neck Tumors: What are the Current Applications?

**DOI:** 10.1007/s11912-024-01546-1

**Published:** 2024-05-23

**Authors:** Po Ling Catherine Chan, Eddy Wai Yeung Wong, Jason Ying Kuen Chan

**Affiliations:** Department of Otorhinolaryngology, Head and Neck Surgery, The Chinese University of Hong Kong, Hong Kong, China

**Keywords:** Robotic surgery, Head and neck surgical oncology, Systematic review, Robotic approaches, Head and neck tumor, Robotic advancement

## Abstract

**Background:**

The journey from radical treatments to the precision of robotic surgery underscores a commitment to innovation and patient-centered care in the field of head and neck oncology.

**Purpose of review:**

This article provides a comprehensive overview that not only informs but also stimulates ongoing discourse and investigation into the optimization of patient care through robotic surgery. The literature on current robotic applications within head and neck region was systematically reviewed.

**Recent findings:**

Thirty-four studies with a total of 1835 patients undergoing robotic surgery in head and neck region were included. Clinical staging, histological types, operative duration, postoperative complications, functional recovery and survival outcomes were compared and evaluated.

**Summary:**

Clinical outcomes have shown promising results and thus the indication on the robotic usage has no longer been limited to oropharyngeal region but from skull base to neck dissection. The latest advancement in robotic surgery further refines the capabilities of surgeons into previously difficult-to-access head and neck regions and heralds a new era of surgical treatment for head and neck oncology.

## Introduction

The realm of head and neck oncology often presents technical challenges for otolaryngologists and head and neck surgeons for its intricate anatomy and the paramount importance of achieving clear resection margins while maintaining the best functional outcomes. The surgical management of head and neck tumors is often the first-line treatment with curative intent unless in the case of specific histological diagnoses or significant resection related functional impairment as this may dictate alternative treatment strategies such as neoadjuvant or radical chemoradiotherapy. While open surgery has traditionally been the cornerstone of head and neck tumor resection, there is a paradigm shift towards minimally invasive surgical techniques in the recent ongoing advancement of robotic surgery and robotic-assisted surgical systems.

Ritter et al. offers a comprehensive overview of recent advancements in the head and neck oncology that spanned the entire spectrum of patient care, from diagnosis to treatment [1].

This review does not confine itself solely to robotic surgery but encompasses a broader scope of progress within the field. Conversely, Boehm et al. wrote a systematic review that delves into the utilization of various robotic platforms across different anatomical regions within head and neck surgery [2••]** It meticulously examines the implications of robotic surgery, including cost-related issues and the current landscape of research and clinical trials, drawing on literature in both English and German.

In this systematic review, we will provide a clinical perspective and analysis of the current applications of robotic surgery for head and neck tumors. Our discussion will extend to include research from China, where robotic surgery has been widely adopted. Despite its prevalence, there has been limited evaluation of these practices when compared to the Western countries. Our paper aims to bridge this gap, offering a critical assessment of the global advancements in robotic surgery in head and neck oncology.

## Methods

To conduct a comprehensive review of the current applications of robotic surgery in the treatment of head and neck tumors, a systematic literature search was performed across several databases, including PUBMED, MEDLINE, and EMBASE. The search was aimed at identifying relevant articles published in both English and Chinese to ensure a broad coverage of the topic. The keywords used for the search were "head and neck surgery," "robotic surgery," and "head and neck tumor." This strategy was designed to capture the most relevant and recent studies that discuss the advancements, applications, and outcomes of robotic surgery in the context of head and neck oncology.

**Fig. 1:**
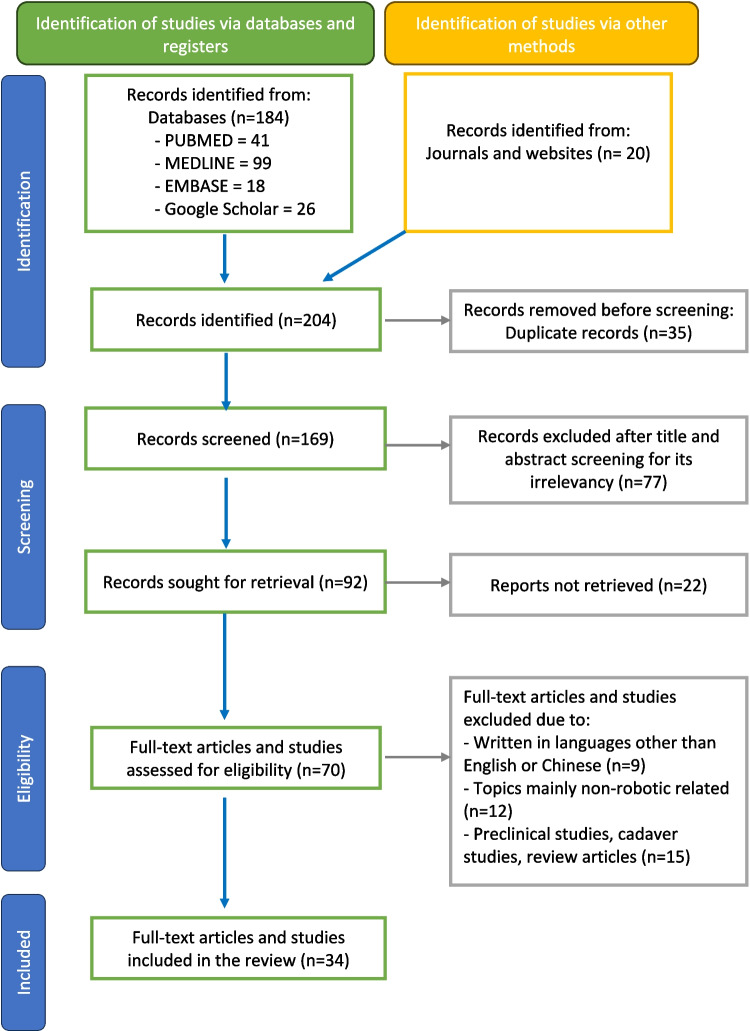
PRISMA (Preferred Reporting Items for Systematic Reviews and Meta-analyses) flowchart

The initial search yielded a total of 184 articles, with 41 articles from PUBMED, 99 articles from MEDLINE, 18 articles from EMBASE, and 26 articles from Google Scholar. An additional of 20 articles were further hand-searched by authors to include the latest publications. To refine the search results and ensure the inclusion of the most pertinent studies, a two-step screening process was employed. First, two independent reviewers conducted a preliminary screening based on titles and abstracts. Studies that were not directly related to the topic and written language that was not in English nor Chinese were eliminated. Following the initial screening, the reviewers further assessed the relevance and quality of the studies. The inclusion and exclusion criteria were designed to select studies that provided significant insights into the applications, techniques, outcomes, and advancements of robotic surgery in treating head and neck tumors. Figure 1 In order to evaluate the clinical outcome of robotic application in head and neck surgery, case reports and clinical trials were included. Studies that were published before 2013, review articles and meta-analysis were excluded. After applying the inclusion criteria and conducting a thorough review, the selection process resulted in 34 articles being identified as highly relevant for this review. Figure 1 presents the PRISMA flowchart to illustrate the methodology for selecting and refining articles, providing the final inclusion of articles for this review. The summary of studies on the robotic application in various clinical diseases is listed in Table 1.

**Table 1:** Summary of Studies

Author (Year of Publication)	City/Country	No	Subsites / Histology (cases)	Gender M:F (total in study)	Mean age (range) in Years	Complications (not stratified by subsites of diseases)	Mean Follow up (months)
Oropharyngeal cancer		Total: 992					
1. Hardman et al.[4] (2022)	Multicentres	259	Tonsil (88) Tongue Base (146) Soft palate (11) Posterior OP wall (14)	222:56	61 (38–93)	Bleeding (8.1%) Fistula (0.7%) Free flap failure (5.4%) Death (1.8%)	NA
2. D’Andrea et al.[10] (2022)	France	53	Tonsil (17) Tongue Base (32) Posterior OP wall (4)	42:11	64.3 (37–87)	Bleeding (11.4%) Fistula (0) Death (3.77%)	NA
3. Meulemans et al.[7] (2021)	Belgium	31	NA	43:18	64.5	Bleeding (11.9%)	17.8
4. Gorphe et al. [11] (2021)	France	50	Tonsil (14) Tongue Base (22) Others (14)	40:10	61.6 (29–80)	Bleeding (10.5%) Fistula (0) Free flap failure (8%) Death (4%)	15
5. Gorphe et al. [12] (2017)	France	20	NA	24:3	62.7 (43–81)	Bleeding (11.11%) Death (0)	NA
6. De Almeida et al. [8] (2015)	Multicentres	364	Tonsil (165) Tongue Base (115) Others (84)	338:72	59.6	NA	20
7. Van Loon et al. [9] (2015)	The Netherlands	18	Tonsil (9) Tongue Base (5) Others (4)	13:5	62.6 (54–77)	Bleeding (11.11%)	33.7
8. Li et al. [6] (2023)	China	83	Tonsil (52) Tongue Base (20) Others (11)	71:12	57	Bleeding (2.41%)	NA
9. Zhang et al. [13] (2022)	China	52	Tonsil (27) Tongue Base (13) Others (12)	43:9	NA	NA	NA
10. Holsinger et al. [14] (2019)	USA and Hong Kong	47	Tonsil (23) Tongue Base (16) Others (8)	39:8	61	Bleeding (4.26%)	NA
11. Fang et al. [15] (2018)	China	13	Tonsil (5) Tongue Base (4) Others (4)	20:10	56 (30–81)	NA	13
12. Chen et al. [18] (2016)	China	2	NA	5:1	53.8 (25–70)	NA	NA
Parapharyngeal Space Tumor		Total:45					
1. Gorphe et al. [12] (2017)	France	1	NA	24:3	62.7 (43–81)	Bleeding (11.11%) Death (0)	NA
2. Fang et al. [15] (2018)	China	7	Pleomorphic adenoma (3) Schwannoma (2) Basal cell adenoma (1) Parapharyngeal space cyst (1)	20:10	56 (30–81)	NA	13
3. Salzano et al. [20] (2022)	Italy	14	Pleomorphic adenoma (14) Size of tumor: 2.5–4.5 cm	5:9	48 (23–74)	NA	minimum FU = 6 months)
4. Wu et al. [19] (2022)	China	23	Pleomorphic adenoma (10) Schwannoma (6) Others (7) Size of tumor: 4.4 cm (2.4–7.5 cm)	13:10	45.3 (25–67)	Postoperative Swelling (17.39%) Bleeding (4.35%) Seroma (13.04%)	NA
Thyroidectomy, lateral neck dissection		Total: 375					
1. Zhang et al. [25] (2022)	China	20	TOTVA: PTC (18) Follicular thyroid carcinoma (1) Benign nodule (1)	0:20	30	Transient RLN (1) Skin flap burn (1) Transient hypocalcemia (4) Mental nerve injury (8)	36
2. You et al. [31] (2021)	South Korea	317	BABA (robotic): PTC (282) Follicular thyroid carcinoma (2) Benign nodule (33)	30:287	40	Seroma (1) Transient RLN (2) Transient hypocalcemia (33) Permanent hypocalcemia (1)	61
3. Cai et al. [26] (2022)	China	10	TORS: lingual thyroglossal duct cyst (10)	6:4	5–44	No complications reported	17
4. Rao et al. [29] (2020)	India	14	TORS lateral neck dissection (14)	12:2	NA	NA	NA
5. Tae et al. [30] (2022)	South Korea	14	PTC (14): • TORS only (10) • TORS + postauricular (4)	3:11	37.3	NA	NA
Laryngeal cancers		Total: 44					
1. Gorphe et al. [12] (2017)	France	5	NA	24:3	62.7 (43–81)	Bleeding (11.11%) Death (0)	NA
2. Fang et al. [15] (2018)	China	6	Supraglottic type: T1 (2); T2 (4)	20:10	56 (30–81)	NA	13
3. Morisod et al. [42] (2018)	Switzerland	2	T2 (1: anterior commissure) T3 (1: left vocal cord)	2:0	70.5 (66–75)	NA	Up to 3 years
4. Meng et al. [41] (2018)	China	7	Supraglottic type: T1 (1) T2 (6)	7:0	63.5 (38–81)	NA	3–20
5. De Almeida et al. [8] (2015)	Multicentres	24	NA	338:72	59.6	NA	20
Hypopharyngeal cancer		Total:63					
1. Gorphe et al. [12] (2017)	France	1	NA	24:3	62.7 (43–81)	Bleeding (11.11%)	NA
2. Fang et al. [15] (2018)	China	4	Pyriform sinus SqCC (2) Pyriform sinus hemangioma (2)	20:10	56 (30–81)	Tracheostomised (0)	13
3. Meng et al. [41] (2018)	China	5	Pyriform sinus SqCC (2) Pyriform sinus hemangioma (3)	3:2	45.4 (30–70)	NA	3–20
4. Park et al. [40] (2017)	South Korea	38	Pyriform fossa (27) Posterior pharyngeal wall (10) Post cricoid (1)	38:0	66.7 (39–88)	Long term tracheostomy (7.9%) Long term tube feeding (2.2%)	Up to 5 years
5. Lorincz et al. [39] (2015)	Germany	5	Pyriform fossa (5)	5:0	64 (57–70)	Bleeding (4%) Temporary Tracheostomy (1) Long term tube feeding (2)	18
6. Liang et al. [43] (2024)	China	1	Pyriform fossa – solitary fibrotic tumor	0:1	78	NA	16
7. De Almeida et al. [8] (2015)	Multicentres	9	-	338:72	59.6	NA	20
Nasopharyngeal carcinoma		Total:86					
1. Tsang et al. [49] (2022)	Hong Kong	31	recurrent NPC T-stage: rT1 (25) rT2 (0) rT3 (6) Approach: TORS only (27) Combined (6)	20:11	55 (29–85)	Bleeding (1) Airway obstruction (1) Palatal fistula (3) Death (2)	38
2. Tsang et al. [50] (2015)	Hong Kong	12	recurrent NPC T-stage: rT1 (8) rT2 (0) rT3 (4) Approach: TORS only (5) Combined (7)	7:5	53.5	NA	28.2
3. Han et al.[51] (2018)	China	10	recurrent NPC T-stage: rT1 (10) Approach: TORS only (2) Combined (8)	7:3	43 (27–65)	Palatal fistula (1) Bleeding (0) Death (0)	6
4. Han et al.[48] (2022)	China	33	Approach: TORS only (18) Combined (13)	20:13	47.9	NA	3–54
Retropharyngeal Lymph node dissection		Total: 100					
1. Ding et al.[55] (2023)	China	68	NPC (28) Thyroid (2) OP (38)	37:31	52.4 (32–69)	NA	Up to 3 years
2. Dabas et al.[54] (2022)	India	10	NPC(1) Medullary thyroid cancer (1) OP (6) Unknown primary (1) Sinonasal carcinoma (1)	7:3	59	Velopharyngeal incompentence (3)	20
3. Ding et al.[53•] (2021)	China	10	NPC (10)	7:3	38	Convert to Open approach (2) Dysphagia (10%)	19
4. Givi et al.[52] (2016)	USA	12	PTC (3) OP (9)	8:4	63	Bleeding (8%) Horner syndrome (16.7%) Aspiration pneumonia (50%)	10
Neoadjuvant chemotherapy with TORS		Total: 130					
1. Wang et al.[59] (2021)	Taiwan	30	OP (7) Larynx (3) HP (20)	28:2	55.9	Long term Tracheostomy (6.7%) Laryngeal preservation rate (96.7%0 VHI-10: 1.5	38.9
2. Sampieri et al.[58•]* (2023)	South Korea	100	Larynx – supraglottis (35) HP- Postcricoid (2) HP- posterior pharyngeal wall (12) HP- Pyriform sinus (51)	97:3	67 (44–83)	Long term Tracheostomy (17%) Long term tube feeding (15%)	24

Appreviations: NA = Data not available; M = Male; F = Female; FU = follow up; NPC = nasopharyngeal carcinoma; RLN = recurrent laryngeal nerve; PTC = papillary thyroid carcinoma; SqCC = squamous cell carcinoma; OP = Oropharynx; HP = hypopharynx; BABA = Bilateral axillo-breast approach; TOTVA (Transoral Transvestibular approach); TORS = Transoral robotic surgery

## Transoral Robotics Surgery (TORS) for Oropharyngeal *Cancer*

In the recent decades, minimal invasive surgery with endoscopic approach has extend the applications in treatment of head and neck tumors. With the emerging of robotic surgery, the innovative technology empowers surgeons to execute more complex operations with unprecedented precision, flexibility, and control, surpassing the capabilities of traditional techniques. Pioneered by O’Malley, Jr. and Weinstein, MD and their team, transoral robotic surgery (TORS) has been a groundbreaking development in head and neck oncology [3]. TORS introduced a novel method for addressing oropharyngeal carcinoma, providing a surgical alternative to conventional chemo-irradiation. In the evaluation of TORS for oropharyngeal cancer treatment, a total of 12 studies were selected. These studies collectively encompass a diverse cohort, with 992 patients. The reported mean age across these studies varied, with some noting an average age of 61 years. Notably, a higher prevalence of oropharyngeal squamous cell carcinoma (OPSCC) was observed in males, with one study documenting a male-to-female ratio of 4:1[4]. The status of Human Papillomavirus (HPV), a recognized prognostic factor in OPSCC, was also examined, revealing a significant incidence of HPV-positive tumors. This aligns with the epidemiological shift observed in OPSCC etiology [5]. That said, three studies included revealed that p16 status did not exert prognostic effect to survival outcome in those patients undergoing TORS [6–8].

Within the analyzed studies, the application of TORS in the treatment of OPSCC predominantly targets early-stage tumors, specifically those classified as T0-T2 [6, 9]. Interestingly, a subset of these studies also encompasses advanced stages of the disease, suggesting a broader potential applicability of TORS beyond early stage of OPSCC [4, 7, 8, 10–15]. Furthermore, the studies under review consistently highlight the impact of surgical margins on patient survival. Both positive margins and close margins are identified as risk factors that compromise local control rate and overall survival [4, 8, 10]. Extranodal extension is another critical factor adversely affecting survival outcomes [10]. This underscores the importance of achieving clear surgical margins and addressing extranodal extension to improve patient prognoses.

Surgical margin, a crucial determinant of oncologic surgery success, exhibited variability across the studies reviewed. Traditionally, a 5 mm distance from the tumor is considered a clear margin. However, ECOG3311 study group stratified risk groups with consideration of close margin as less than 3 mm, reporting a favourable oncologic outcome and functional outcomes with primary transoral surgery for HPV positive OPSCC [16]. Hardman et al. found that patients with positive margins and those with margins over 1 mm had two-year local control rates of 48.2% and 80.9%, respectively [4]. Meulemans et al. defined a clear margin as 2 mm, resulting in a local control rate of 91.7% over two years [7]. These findings support the re-definition of margin clearance, particularly for TORS in HPV related OPSCC, to align with de-escalation management in intermediate risk group of patients.

Post-TORS hospital stay durations also varied in studies evaluated, with the median length of stay ranges from 1.5 days to 25 days. Operating time was crucial for surgical planning and resource allocation. Two studies separated docking time from operative time, with an average of 12–20 min for docking and setup [7, 12]. The average operating time in the studies were between 34.7 and 115 min, except Grophe’s team being 574 min including free flap reconstruction [6, 9, 11].

The overall success rate of TORS in these studies is shown to be high, with up to 89% of surgeries completed without major complications [4]. However, postoperative bleeding remains the most common complication, with reported rates ranging from 2.41% to 11.9% [4, 6, 7, 9–11, 14]. Prophylactic transcervical ligation of external carotid artery during neck dissection has been proposed to reduce severe postoperative haemorrhage [17]. In the immediate postoperative period, a significant proportion of patients between 33% and 75.4% require tracheostomy [4–7, 10, 11, 14, 18]. This is often a temporary measure to secure the airway until oropharyngeal airway swelling subsides and normal breathing is possible. One study reported a 100% decannulation rate, with the median time to removal of the tracheostomy tube being 12.5 days postoperatively, suggesting that most patients can expect to have their tracheostomy tubes removed within two weeks after surgery [4, 11]. Despite the high rate of initial tracheostomy, long-term dependence on tracheostomy is relatively low. Some studies have reported that about 8–10% of patients remain dependent on a tracheostomy at the one-year [4, 10, 13].

Moreover, beyond the surgical and pathological parameters, patient-related factors such as smoking status and older age have emerged as significant risk factors impacting survival [6, 8]. These findings collectively highlight the multifaceted nature of prognostication in OPSCC, where tumor biology, surgical precision, and patient-specific factors intertwine to influence outcomes.

These findings underscore the importance of patient selection, surgical expertise, and postoperative care in the successful application of TORS. As such, a comprehensive approach that considers these diverse elements is crucial for optimizing treatment strategies and improving survival rates in patients undergoing TORS for OPSCC.

## Transoral Robotic Surgery Approach Towards Parapharyngeal Space (PPS) Tumor

Transoral Robotic Surgery (TORS) has emerged as a promising minimally invasive approach for accessing and resecting tumors in the parapharyngeal space (PPS), a challenging anatomical area due to its deep location and proximity to vital neurovascular structures.

Four articles focusing on TORS for PPS tumors were selected to highlight its feasibility, efficiency and safety profile, offering a new horizon in head and neck surgery. A collective total of 45 patients underwent TORS for PPS tumors, with a notable male predominance up to 8 folds [12, 15, 19, 20]. The patient age spanned from 30 to 62.7 years, with the majority of cases involving benign histologies such as pleomorphic adenoma or schwannoma. Within this patient cohort, two instances of margin involvement were reported, characterized by the rupture of the tumor capsule [15, 20]. Nevertheless, an impressive 93% of cases achieved complete tumor resection with an intact capsule [20]. Follow-up periods, with a median duration of 6 to 13 months, did not reveal any recurrence of the disease [15, 20]. However, given the benign nature of these tumors, a longer follow-up is essential to thoroughly assess the long-term efficacy of TORS in ensuring complete tumor clearance. The average hospitalization was about 3 to 9 days, reflecting a relatively brief recovery period [15, 19, 20]. Operative times were efficient, ranging from 40.7 to 118.2 min, for reported mean tumor size up to 7.5 cm in largest diameter [19]. Only one study separately reported an average docking time of 12 min [12]. Intraoperative blood loss during the procedures was reported to be between 15.8 and 141.9 ml by its mean volume [12, 15, 19].

Complications associated with TORS for PPS tumors were generally minor, with the most common postoperative issues being swelling, pain, and seroma formation. The rate of postoperative bleeding was low, reported between 4.35% and 11.11% [12, 19]. There was no instance of tracheostomy or tube feeding required after surgery, indicating a favorable postoperative course with respect to airway management and nutritional intake. These findings suggest that TORS is a viable and relatively safe surgical option for PPS tumors, with potential advantages in terms of reduced operative morbidity and hospital stay.

## Robotic Thyroidectomy and Lateral Neck Dissection

The advent of robotic thyroidectomy represents a significant shift in the surgical management of thyroid cancer. Traditionally, thyroidectomies have been performed through an open approach in the anterior neck. This conventional method, while effective, often results in a visible scar, prompting more researches on less invasive techniques with better cosmetic benefits. In 1997, Huscher proposed thyroidectomy via endoscopic means [21]. Since then, remote access approaches for endoscopic thyroidectomy have drawn more researchers’ interests [22]. These methods aim to minimize invasiveness and improve cosmetic outcomes by avoiding a collar scar in the neck. Despite these advantages, the endoscopic approach has been associated with longer operative times and a steep learning curve [23]. In recent years, the emergence of robotic-assisted remote access surgery for thyroidectomy has marked a new chapter in this surgical field. Robotic systems offer enhanced dexterity, precision, and three-dimensional visualization, potentially overcoming some of the limitations of endoscopic techniques.

While Tae et al. described different approaches of robotic and endoscopic thyroid surgery including the transoral endoscopic thyroidectomy vestibular approach (TOETVA) with CO2 insufflation, Chan et al. further demonstrated a feasibility cadaveric trial using the new generation of flexible single port system- the da Vinci SP (Intuitive, Surgical Inc., Sunnyvale, CA, USA [23, 24]. The advantage of using the single port cannula from da Vinci SP allows the manipulation of three instruments in addition to a camera within a 2.5 cm single cannula, without severing mental nerves in trans-vestibular route, surpassing the bulky instrumentation from the current da Vinci Si and Xi robotic systems [24, 25]. That said, transoral robotic surgery using da Vinci Si surgical system has also been implemented in paediatric population for lingual thyroid and thyroglossal duct cyst excision [26]. It was reported that the docking time and operative time were short and efficient, averaging 15.5 min and 17.6 min respectively. This expedited procedure was complemented by minimal intraoperative blood loss, favourable postoperative outcomes including swift resumption of diet, null airway complications, and voice quality preservation, thereby suggesting TORS as a viable and safe surgical option even in paediatric population [26].

Vanermen et al. conducted an extensive systematic review on robotic thyroid surgery, encompassing a total of 14,433 patients in 68 studies, to evaluate various outcomes of robotic thyroid surgery [27•]*. Reviewing the operative times, postoperative pain, duration of hospital stay, and complication rates in each approaches, this comprehensive analysis support the notion that robotic thyroidectomy is not only comparable to conventional open surgery for its feasibility and safety profile but also provides superior cosmetic satisfaction and an improved quality of life [27•]*.

With experiences from robotic assisted thyroidectomy and central neck dissection, the exploration of surgical territories has expanded from central to lateral neck dissection. Wood’s review summarised the current state of art on remote access lateral neck dissection for thyroid cancer, covering endoscopic, video-assisted and robotic means [28••]**. Focusing on robotic approaches, various methods have been explored, including transaxillary, bilateral axillo-breast (BABA), retroauricular facelift, and transoral vestibular (TOTVA) approaches. The review meticulously evaluates the indications, contraindications, outcomes, complications, and advantages and limitations of each approach, underscoring the challenges to broader application, including strict patient selection criteria, a limited case volume, a steep learning curve, technical complexities, and a scarcity of expertise [28••]**. Rao et al. share their experience with the retroauricular approach, detailing the procedure across 14 cases of robotic selective neck dissection [29].

Tae et al. present a comparison between transoral robotic selective neck dissection with and without a postauricular incision, outlining the operative steps for both methods in fourteen patients [30]. This study demonstrates the feasibility of transoral robotic selective neck dissection for levels III, IV and V dissection without need of postauricular incision under gas insufflation. On the contrary, level II lymph node dissection was shown to be challenging due to limited surgical view and instrument access, prompting the addition of a gasless postauricular approach to enhance access to level II lymph nodes [30]. While both methods were able to achieve similar number of lymph node removal in central neck dissection, combined transoral and postauricular access in tackling lateral neck dissection has shown to have higher number of lymph node removal (38.3 ± 8.5 VS 23.1 ± 9.4) and positive lymph node detection (8.3 ± 2.5 VS 2.9 ± 1.9), suggesting a better surgical clearance yet unaffecting on recurrence rate upon follow up in both groups [30]. Despite higher lymph node clearance on combined approach, the mean operative time was lengthened from 299 min in sole transoral approach to 431 min in combined approach. The rate of transient recurrent laryngeal nerve palsy was reported 3.7% but none was reported on mental nerve injury. Three patients (21.5%) had transient hypoparathyroidism and one patient had postoperative chyle leak that was managed conservatively.

The exploration of robotic techniques for thyroidectomy and neck dissection is demonstrating promising outcomes in terms of feasibility, safety, and postoperative recovery. The innovative use of combined approaches to overcome anatomical challenges, particularly for level II lymph node dissection, underscores the adaptability and potential of robotic surgery in the management of thyroid cancer. Nevertheless, it is crucial not to sacrifice the thoroughness of tumor removal, jeopardize the integrity of recurrent laryngeal nerve and the overall oncological results in favor of a minimally invasive and scarless approach in robotic thyroidectomy.

The consensus across the studies recommends that benign nodules smaller than 6 cm, intermediate neoplasia or follicular neoplasm, and T1 and T2 differentiated thyroid carcinoma are suitable for robotic approaches [31]. Conversely, patients with a high Body Mass Index (BMI), symptoms of thyroiditis or Grave’s disease, initial tumor sizes larger than 4 cm, or those diagnosed with poorly differentiated or undifferentiated carcinoma are better treated by traditional open surgical methods [32]. Moreover, the financial implications of robotic surgery remain unexplored. The cost analysis comparison is essential, illustrating the importance of judicious patient selection, ensuring that the benefits of employing these advanced technologies are outweighed its economic impact and resources allocation.

As robotic technology continues to evolve, it has become an attractive option for patients seeking effective treatment for thyroid cancer with the added benefit of minimal scarring and enhanced postoperative recovery. Further research and clinical trials will be needed to investigate the pros and cons of different approaches, to evaluate on the need of gas or gasless procedure and to validate the safety, efficacy, oncological outcomes and long term outcomes using robotic assisted application in thyroid surgery and neck dissection.

## Transoral Robotic Surgery for Laryngeal and Hypopharyngeal Tumors

The landscape of laryngeal and hypopharyngeal cancer treatment has undergone significant evolution over the past few decades. Traditionally, the management of early glottic cancer has been oscillating between open surgery and chemoradiation, with an emphasis on laryngeal preservation strategies emerging in the 1990s for locally advanced laryngeal carcinoma [33, 34]. This shift of laryngeal preservation management was also observed in hypopharyngeal cancer in both early and advanced stage of the disease, attempting to maintain patient’s voice and swallowing functions for better quality of life [35]. Concurrently, with the advancement in microlaryngeal surgery and application of laser therapy, transoral Laser Microsurgery (TLM) marked a pivotal moment in the surgical treatment of early glottic and hypopharyngeal cancer, suggesting a favourable alternative option for these group of patients [36]. Furthermore, taking advantages of narrow band imaging (NBI) technique in oesophagogastroduodenoscopy (OGD), earlier detection of hypopharyngeal tumors, which historically was often diagnosed at advanced stages, has gained widespread recognition [37]. The advent of Endoscopic LaryngoPharyngeal Surgery (ELPS) provided a new avenue for addressing these cancers at an earlier stage, offering patients the benefits of minimally invasive surgery and the potential for improved functional outcomes [38•]*.

Leveraging the precision of laser technology and optical diagnosis by NBI, TORS represented the next frontier in the evolution of laryngeal and hypopharyngeal cancer surgery. Building on the principles of minimally invasive surgery, TORS introduced the advantages of robotic technology, including enhanced dexterity, precision, and visualization. Researchers and clinicians began to investigate the potential of TORS for the treatment of early glottic and hypopharyngeal cancers, motivated by the goal of reducing the toxicity associated with radiotherapy while achieving effective tumor clearance.

In this review, a comprehensive analysis of eight studies encompassing 44 patients with laryngeal carcinoma and 63 patients with hypopharyngeal carcinoma reveals a notable male predominance, with average ages spanning from 56 to 70.5 years [8, 12, 15, 39–43]. Predominantly, these studies focused on T1 and T2 stages of cancer, although one study also included patients locally advanced hypopharyngeal carcinoma, providing valuable insights in long-term oncological and functional outcomes [40]. A majority of the studies reported successful attainment of clear surgical margins in TORS procedures for both laryngeal and hypopharyngeal carcinoma [41, 42]. However, Park et al. noted a 21% (8/38) incidence of positive margin in their series, while Lorincz et al. reported a 20% (1/5) rate of close margins in their cohort of early stage T1/T2 hypopharyngeal cancer [39, 40].

The manipulation and execution of TORS was generally smooth and efficient, with docking time of 12–31 min and operative time averaging 36.4 min for laryngeal cancer and 37.5 min for hypopharyngeal cancer treatments [39, 41]. Intraoperative blood loss was consistently minimal, with volumes ranging from 7.5 to 30 ml [15, 41, 43]. Despite the focus on TORS for laryngeal and hypopharyngeal cancers, only two studies delved into acoustic analysis on voice’s quality, revealing that while the fundamental frequency of the voice remained similar to control groups, there was an increase in jitter and shimmer variation in TORS group [40, 42]. In terms of swallowing function, studies reported promising results with most patients resuming a normal oral diet within an average of 15.9 days postoperatively [39, 40, 42, 43]. Park et al. provided a long-term outcome of early and advanced stages of hypopharyngeal carcinoma, demonstrating a 100% five-year survival rate for early stage hypopharyngeal cancer treated with TORS, and a 68.6% disease-free-survival rate for advanced stages. The overall survival and local control in TORS for hypopharyngeal cancer were reported at 44.7% and 88.9% over five years, respectively, which were comparable to conventional treatment such as TLM, radiation and chemoradiation [40, 44, 45].

TORS offered a minimally invasive alternative that allowed for targeted tumor resection with reduction of detrimental morbidity when compared with total laryngectomy with or without pharyngectomy in the case of laryngeal and hypopharyngeal carcinoma. This technique seems to provide a viable option for patients, demonstrating promising oncological and functional outcomes. When exploring the capabilities of TORS in the treatment of laryngeal and hypopharyngeal cancers, the focus remains on optimizing patient outcomes—balancing the need for oncological clearance with the preservation of voice, swallowing, reduction in adverse effects from multimodalities treatment and overall quality of life.

## Robotic Nasopharyngectomy and Retropharyngeal Lymph Node Dissection

Nasopharyngeal carcinoma (NPC) is notably prevalent in Southern China and is traditionally treated with primary radical radiotherapy in early stages and chemoradiation in advanced stages. Long-term survivors of NPC undergo regular surveillance due to the possibility of recurrence. Constrained by the prior irradiation, when NPC does recur, open surgery over mid-face with various approaches has made the disease salvageable and control. Yet, access and visualisation to the nasopharynx remains difficult and fine manipulation was also limited by instrumentation and confined space [46]. With the introduction of robotic surgery, TORS nasopharyngectomy for local recurrence has made possible, with initially via split palate approach, reducing postoperative morbidity with great dissection workspace, manipulation, clear 3D vision and resection [47].

In this review, four studies were selected, with a total of 86 patients undergoing TORS nasopharyngectomy with trans-palatal approach or combined nasoendoscopic-TORS approach. Both Tsang’s and Han’s groups provided the long term data of 5 years and 3 years survival outcome respectively [48, 49]. Notably, male predominance across the studies was observed with about 1.7:1 male to female ratio. The average age of patient at the time of operation was 49.9. Patient were usually diagnosed with an interval from primary NPC by 32–53.5 months in Tsang’s studies [49, 50]. Among the 86 patients, five cases were reported to have positive margin involvement (two cases with R1 and three cases with R2 margin), which required reoperations or adjuvant therapy [51]. The majority of NPC recurrence was in early stage. Locally advance stages (rT3) was also included for analysis [49, 50]. The mean operative time was reported with an average of 126.2 to 227 min. Intraoperative blood loss was variable, as low as 5.5 ml, with one study reporting an average of 200 ml. These studies stated the fact of mild complication rate in TORS in majority of patients. However, a few patients suffered from nasopalatal fistula, especially with split palatal approach. A significant highlight in Han’s and Tsang’s work are the long term survival outcomes in TORS nasopharyngectomy for recurrence of NPC. While positive margin was still the key negative prognosticator for survival outcome, the reported 3- and 5-year overall survival rate was 92.9% and 55.7%; local control rate was 91.7% and 83.2%, respectively [48, 49]. Furthermore, Tsang’s group provides a valuable guidance on the indications and contraindications for TORS suitability in recurrent NPC, maximizing benefits through careful patient’s selection while minimizing risks [49]. The suggested indications were a small nasopharyngeal tumor with minimal lateral extension beyond lateral pterygoid plate, at least 5 mm from internal carotid artery and tumor without gross invasion into clivus marrow [49, 51].

These findings suggested TORS being a feasible and safe option comparing to an open surgery procedure in the case of NPC recurrence, offering an alternative management with mild complication profile, when option of irradiation is obsolete. Nonetheless, the limited number of studies highlight the need for further research to assess the long-term outcomes in the treatment of recurrent NPC.

Furthermore, retropharyngeal lymph node often serves as the initial site of sentinel metastatic spread from nasopharyngeal carcinoma, leading to N1 stage disease. This is consistent with patterns observed in other head and neck tumor, such as oropharyngeal cancer, where involvement of retropharyngeal lymph nodes typically necessitates radical radiotherapy due to their deep-seated location, making them challenging targets for conventional neck dissection. That said, Transoral Retropharyngeal Neck Dissection (TORPND) is being explored as a potentially curative oncological treatment option, especially in the case of recurrence or with previous irradiation.

Four studies were selected for analysis, encompassing a total of 100 patients to assess the feasibility and safety profile of TORPND [52, 53•, 54, 55]. The primary tumor sites included oropharyngeal cancer (53 cases) and nasopharyngeal cancer (39 cases), with a smaller number originating from papillary thyroid carcinoma (5 cases), medullary thyroid carcinoma, carcinoma of unknown primary and sinonasal carcinoma. Across these studies, a slight male predominance was noted, with one study by Ding et al., involving 68 cases of TORPND, showing a similar gender ratio. Patient ages ranged from 38 to 63 years on average. The reported average operative time varied significantly, from 28.5 to 297 min, and the length of hospital stay ranged from 1.8 to 8.52 days. Average blood loss during these procedures was between 40 to 150 ml. While Ding’s study did not detail the complication profile, the other three articles reported an 8% bleeding rate, 10% incidence of dysphagia, 40% velopharyngeal incompetence lasting up to three weeks, and a risk of aspiration pneumonia postoperatively in up to 50% of cases [52, 53•, 54, 55]. Some patients required tube feeding for 5 to 12 days postoperatively.

Ding et al. provided 3 years survival outcome analysis after TORPND, with overall survival rates of 91.18%, 85.29%, and 70.59% observed at 1-, 2-, and 3-year intervals, respectively [55]. Other studies, with a median follow-up of 10–20 months, a 92% local survival rate and a 10% rate of cervical recurrence were reported [52, 53•, 54]. Prognostically, the presence of retropharyngeal nodes was associated with a negative impact on survival compared to similar primary sites of head and neck tumors without retropharyngeal nodal metastasis [54, 55]. Additionally, nodal metastasis larger than 6 cm and the presence of lymphovascular invasion were identified as factors adversely affecting patient prognosis [55].

In short, TORPND presents a promising surgical approach for the management of retropharyngeal lymph node metastasis in head and neck cancers, its safety profile and long term outcome warrant further investigation. We highlight the need for careful patient selection. Scenarios of extranodal extension (ENE) and its relations in proximity with carotid arteries were not investigated. Nevertheless, the preliminary data suggest potential benefits in terms of survival and being a feasible alternative in particular for case of salvage dissection.

## The Role of Neoadjuvant Chemotherapy (NAC) Combining with Transoral Robotic Surgery

The role of neoadjuvant chemotherapy (NAC) prior to Transoral Robotic Surgery (TORS) in advanced stage of oropharyngeal cancer has been under investigation for its capacity to de-intensify tri-modality treatment regimens, thereby potentially reducing treatment-related toxicity while enhancing both functional and survival outcomes for patient [56, 57].

In this review, two studies, including 130 patients receiving TORS with long term results, were selected to underscore the potential for achieving optimal oncological and functional outcomes in patients with advanced-stage of head and neck cancer [58•, 59]*. The application of NAC before TORS has been observed to lead to a complete response in the primary tumor site, regional metastasis or both, in one-third of patients, as evidenced by post-TORS pathological assessments. Furthermore, patients who partially responded to NAC experienced a downstaging in clinical staging, enabling a more precise and effective surgical resection, in more than half to three-quarters of cases in the two studies [58•, 59]*. Sampieri’s group, observing both stage III and IV supraglottic and hypopharyngeal cancers treated with NAC and TORS, has shown satisfactory postoperative recovery and functional outcomes, in which 15% of patients did not require prophylactic tracheostomy and most of tracheostomised patients were decannulated at a mean of 12.59 days [58•]*. Only less than one-fifth of patients (17%) were required to maintain a tracheostomy at 6-month postoperatively. Similarly, 15% were gastrostomy dependent in 6 months after surgery. In Wang’s group with 30 cases, with majority of hypopharyngeal cancers, has demonstrated encouraging functional outcomes with 100% resumption of oral diet and 93.3% tracheostomy-free rate [59]. The measured voice handicap index-10 (VHI-10) was 1.5 on average, which has provided an excellent result on patient’s perspective on functional outcomes. That said, Wang’s results include both early and advanced stages of the diseases and only one-third of the cases were receiving NAC prior TORS. The potential benefit of NAC seems promising in tumor shrinkage and high margin-free resection. Yet, it was reported that the effects on organ preservation rate, functional and survival outcomes were not statistically significant [59]. Evidenced further reinforced in Sampieri’s group that the negative prognosticator factors were stage IV disease at presentation, presence of lymphovascular invasion and receiving three cycles of NAC over two cycles, scrutinizing the effects of NAC prior to TORS on head and neck cancer [58•]*.

Nevertheless, the promising findings in complete response rate, long term functional and survival outcomes, and organ preservation rates advocate for the continued exploration and optimization of this treatment approach with judicious patient selection in the management of advanced head and neck cancers.

## Currently Advancement on Different Robotic Systems

The field of robotic surgery in head and neck applications, initially pioneered by the da Vinci Surgical System from Intuitive Surgical, has been introduced with newer robotic systems to accommodate the current challenges in the complicated anatomy of head and neck regions.

Versius Robotic Platform, CMR Surgical, provides multiport design with a smaller bedside footprint, individual portal robotic arms, and open console design to allow comfortable ergonomic posture in the surgery. A preclinical cadaver TORS trial has demonstrated its safety and feasibility on three index transoral procedures, including lateral oropharyngectomy, tongue base mucosectomy and partial supraglottic laryngectomy [60].

Symani Surgical System, MMI, Pisa, Italy, attained CE certification, focusing on robotic assisted microvascular procedures, which could enhance surgeons’ precision and motion stability in addition to reducing tissue damage during manipulation [61].

The OTTAVA™ System, Johnson & Johnson, and Hugo™ RAS system, Medtronics aim to improve surgeon’s experience by introducing adjunct on their robotic system. OTTAVA™ integrates four robotic arms into the standard operative table to provide flexibility for repositioning patients during the surgery [62]. Hugo™ RAS system introduced Touch Surgery™ ecosystem to capture surgical data and provide feedbacks for skill enhancement and consistency [63].

Similarly, there are emerging companies developing robotic systems on various applications in China in the recent decade. TOUMAI endoscopic surgical system from Shanghai MicroPort MedBot Co., was the first commercialised four arm surgical robot developed in China, aiming to provide minimally invasive surgery at lower cost with wider spread of applications [64]. Edge Medical Robotics, ShenZhen China, has developed a single port robotic surgical system(SP1000) and demonstrated feasibility test on human trial for hysterectomy [65]. Sentire Endoscopic Surgical system, Cornerstone Robotics aims to forge accessibility in robotic surgery around the world with affordable cost and high precision in surgery [66]. Agilis Robotics from Hong Kong, targets on endoluminal surgery by developing fully flexible instruments with small workspace [67].

Moving forward, the future of robotic development should embrace a suite of advanced features, including flexible robotic system, enhanced visualisation, augmented reality, haptic feedback and the incorporation of real-time learning algorithms powered by artificial intelligence. These innovations are set to expand the boundaries of robotic applications in future. Together, these emerging robotic systems from all parts of the world have provided alternative choices for future robotic head and neck surgery. With their advanced capabilities to improve surgical experience, ergonomics, economic burden and patient’s outcomes, they help to transform the treatment landscape in robotic applications for head and neck surgical oncology, offering more efficient, cost effective, precise and less invasive treatment options.

## Conclusion

With these emerging technologies and broad applications on head and neck surgical oncology, it is imperative to critically assess the efficacy, safety, and functional outcomes in the long run. This review has highlighted the current trends in robotic head and neck surgery, discussed the implications of these advancements for clinical practice, and considered the future trajectory of robotic interventions in the management of head and neck tumors. The selected articles encompass a wide range of robotic applications in various anatomical pathologies within the head and neck region. This diverse collection of studies provides a concise overview on clinical perspectives and offers insights into the state-of-the-art applications of robotic surgery in treating head and neck tumors.
